# c.464A>G variation in the *GJB2* gene is detected in a Han Chinese family

**DOI:** 10.1002/ccr3.1184

**Published:** 2017-09-15

**Authors:** Gang‐Hua Zhu, Hong‐Ying Shu, Hai‐Yan Zhou, Yong Chen, Fei Zhou, Bin Ni, Wanqin Xie

**Affiliations:** ^1^ Department of Otolaryngology‐Head and Neck Surgery The Second Xiangya Hospital Central South University Changsha Hunan 410011 China; ^2^ Key laboratory of Genetics and Birth Health of Hunan Province Family Planning Research Institute of Hunan Province Changsha Hunan 410126 China; ^3^ Department of Otolaryngology The Third Hospital of Changsha Changsha Hunan 410015 China

**Keywords:** GJB2, hearing loss

## Abstract

We report two heterozygous carriers of c.464A>G variation in the *GJB2* gene in a Chinese pedigree. The proband with hearing loss most likely inherited the c.464A>G variation from his mother who also carries heterozygous c.79G>A variation and has normal hearing. The pathological significance of c.464A>G variation remains to be determined.

## Introduction

The *GJB2* gene encodes the connexin 26 (CX26) protein, which is a major structural component of gap junction channels between cells, and plays a vital role in hearing physiology 1. To date, more than 150 different variants have been reported in the *GJB2* gene 2, of which many have been identified as pathogenic (e.g., c.35delG, c.235delC) or benign mutations in nonsyndromic hearing loss based on clinic and genetic evidence. However, little is known about those rare alleles of *GJB2* pertaining to their clinic implications due to lack of documentation. Here, we report two heterozygous carriers of c.464A>G variation (dbSNP ID: rs776335087) in the *GJB2* gene in a Han Chinese family. Our case report may provide information to clinicians and genetic counselors.

## Case Report

**Figure 1:**
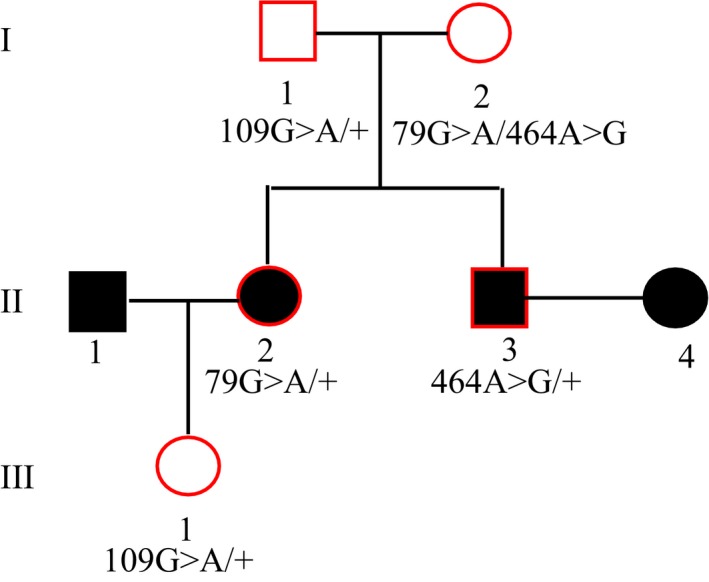
Pedigree of a three‐generation Han Chinese family. The family members who provided blood samples for study are marked with red box and their hearing phenotypes are based on self‐report. The genotypes of *GJB2* of all participants are deduced based on Sanger sequencing results.

A three‐generation Han Chinese family was referred to our institute for genetic counseling (Fig. 1). The young couple, patients II‐3 and II‐4 presented with severe hearing loss and were unable to communicate orally. According to their medical records, the possibility of drug‐induced deafness cannot be excluded. Prior to their visit to our institute, patients II‐3 and II‐4 received targeted gene sequencing in a local genetics hospital. In terms of the report, the diagnostic panel was designed to target exons of 143 genetic deafness‐associated genes, six deafness‐associated mitochondrial DNA regions, and three microRNAs (Tables S1–S3). Targeted sequencing reveals that patient II‐3 is heterozygous for c. 464A>G variation in *GJB2*, and patient II‐4 is heterozygous for both c.235delC and c.176‐191del16 mutations in *GJB2*. No other mutations were detected by the diagnostic panel.

**Figure 2:**
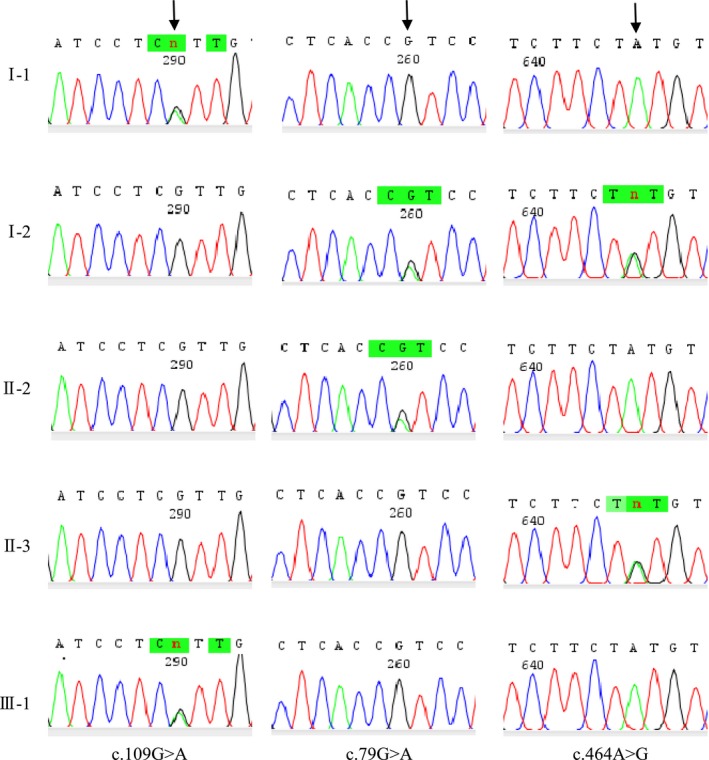
Sanger sequencing reveals multiple variations in *GJB2* in the Chinese pedigree. Chromatograph traces show that individuals I‐1 and III‐1 are heterozygous for c.109G>A variation, and that I‐2 and II‐2 are heterozygous for c.79G>A variation, and that I‐2 and II‐3 are heterozygous for c.464A>G variation.

To find out whether c.464A>G variation in patient II‐3 is a *do novo* mutation or parentally inherited, we recommended Sanger sequencing of exons of the *GJB2* gene for the family. Written informed consent was received from the family and five members (I‐1, I‐2, II‐2, II‐3, and III‐1) provided periphery venous blood samples for analysis. Based on sequencing results (Fig. 2) and the pedigree, the genotypes of all participants are deduced (Fig. 1), and we consider that patient II‐3 most likely inherited the c.464A>G allele from I‐2, his mother.

## Discussion

Mutations in the *GJB2* gene are the most common causes for autosomal recessive nonsyndromic hearing loss (NSHL) 3, 4. In the study by Zheng et al. which involved 1067 Han Chinese subjects, mutations in the *GJB2* gene are responsible for approximately 34.96% of NSHL, and c.235delC is the most frequently observed pathogenic mutation 5. In this report, multiple variations in the *GJB2* gene have been detected in a Chinese family. ClinVar records (https://www.ncbi.nlm.nih.gov/clinvar) show that c. 79G>A variation is generally benign, whereas c.109G>A variation is pathogenic or likely pathogenic. In a recent study, homozygosity of c.109G>A variation is reported to associate with a broad spectrum of hearing phenotypes in Chinese, which can be mild to profound hearing loss or totally normal hearing 6. In this study, both individuals I‐1 and III‐1 are heterozygous carrier of c.109G>A variation and have normal hearing based on self‐report.

Exome Aggregation Consortium has deposited c.464A>G variation of the *GJB2* gene in its online database (ExAC, http://exac.broadinstitute.org/), with one c.464A>G allele being detected among a total of 121,052 alleles from subjects representing diverse ethnicities, equaling to an allele frequency of 8.26 × 10^‐6^. Here, we observed two heterozygous carriers of c.464A>G allele in a Chinese family namely the proband and his mother. The proband's mother also carries c.79G>A variation and exhibits normal hearing. To the best of our knowledge, this is the first report of c.464A>G variant of *GJB2* in Han Chinese. However, our findings are far insufficient to link c.464A>G allele with hearing phenotype and more pedigrees carrying this allele are needed to address this issue.

**Figure 3:**
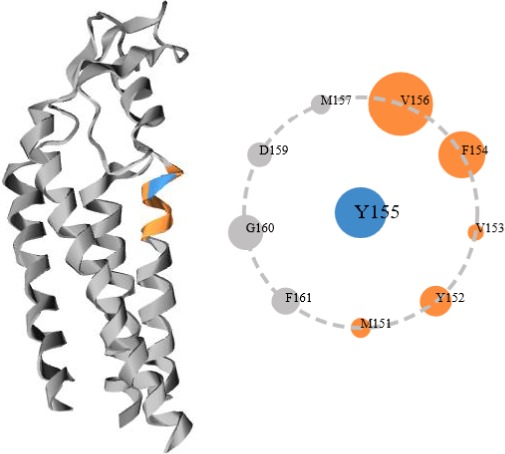
3D structure of GJB2 protein with tyrosine 155 highlighted. Tyrosine 155 residue (blue) localizes in the C‐terminal within a helix structure (yellow). The ring shows neighbor residues that are in immediate atomic contacts with tyrosine 155. The images were generated using Rajini (http://mbgroup.mrc-lmb.cam.ac.uk/).

The c.464A>G variation in the *GJB2* gene leads to substitution of tyrosine 155 by cysteine in its encoding protein. With the well‐annotated 3D structure of GJB2 protein (http://www.ebi.ac.uk/pdbe/entry/pdb/5ER7), it is easy to know that tyrosine 155 localizes in the C‐terminal within a helix structure containing amino acid residues 136–156, where tyrosine 155 is engaged with multiple neighbor residues via immediate atomic contacts (Fig. 3). We speculate that cysteine substitution may interrupt the residue interactions that tyrosine 155 holds. However, it is currently unknown whether the substitution results in a loss‐of‐function or even pathogenic protein. It will be an interesting study to test this mutant in cell line models in the future.

Collectively, our case report shows two heterozygous carriers of c.464A>G variation in the *GJB2* gene, and the pathological significance of c.464A>G allele remains to be ascertained.

## Conflict of Interest

The authors have no conflict of interest to declare.

## Authorship

GHZ: designed the study and analyzed data. HYS: performed experimental studies. HYZ: performed experimental studies. YC: designed the study and interpreted data. FZ: performed physical examination. BN: acquired samples. WQX: analyzed data, made figures, and wrote the manuscript.

## Supporting information


**Table S1**. List of genetic deafness‐associated genes in targeted gene sequencing.
**Table S2**. List of deafness‐associated mitochondrial DNA regions.
**Table S3**. List of deafness‐associated microRNAs.
