# Supplementary material for: c.464A>G variation in the GJB2 gene is detected in a Han Chinese family
Source: Clin Case Rep. 2017 Sep 15;5(11):1785–8. doi: 10.1002/ccr3.1184 (PMC5676280; doi:10.1002/ccr3.1184)
Supplement: Supplementary file 1 — Table S1. List of genetic deafness‐associated genes in targeted gene sequencing. Table S2. List of deafness‐associated mitochondrial DNA regions. Table S3. List of deafness‐associated microRNAs. [file CCR3-5-1785-s001.docx]

Table S1. **List of genetic deafness-associated genes in targeted gene sequencing**

|  | | | | | | | | |
| --- | --- | --- | --- | --- | --- | --- | --- | --- |
| ACTG1 | COL2A1 | ECM1 | GATA3 | IL13 | MSRB3 | PCDH15 | SEC23A | TIMM8A |
| ALX3 | COL4A3 | EDN3 | GIPC3 | ILDR1 | MYH14 | PDZD7 | SEMA3E | TJP2 |
| BSND | COL4A4 | EDNRB | GJB2 | KARS | MYH9 | PNPT1 | SERPINB6 | TMC1 |
| CABP2 | COL4A5 | ELMOD3 | GJB3 | KCNE1 | MYO15A | POLR1C | SIX1 | TMIE |
| CCDC50 | COL4A6 | ESPN | GJB6 | KCNJ10 | MYO1A | POLR1D | SIX5 | TMPRSS3 |
| CDH23 | COL9A1 | ESRRB | GPR98 | KCNQ1 | MYO3A | POU3F4 | SLC17A8 | TNC |
| CEACAM16 | COL9A2 | EYA1 | GPSM2 | KCNQ4 | MYO6 | POU4F3 | SLC26A4 | TPRN |
| CHD7 | CRYM | EYA4 | GRHL2 | KRT9 | MYO7A | PROK2 | SLC26A5 | TRIOBP |
| CIB2 | DFNA5 | FGF3 | GRXCR1 | LAMA3 | NDP | PROKR2 | SMPX | TRMU |
| CLDN14 | DFNB31 | FGF8 | HARS | LARS2 | NF2 | PRPS1 | SNAI2 | TSPEAR |
| CLPP | DFNB59 | FGFR1 | HARS2 | LHFPL5 | OTOA | PTPRQ | SOX10 | USH1C |
| CLRN1 | DIABLO | FGFR3 | HGF | LOXHD1 | OTOF | RDX | STRC | USH1G |
| COCH | DIAPH1 | FLNA | HMX1 | LRTOMT | P2RX2 | RPGR | TBC1D24 | USH2A |
| COL11A1 | DIAPH3 | FOXI1 | HOXA2 | MARVELD2 | PABPN1 | SALL1 | TCOF1 | WFS1 |
| COL11A2 | DSPP | FREM1 | HSD17B4 | MITF | PAX3 | SALL4 | TECTA |  |

|  | |
| --- | --- |
| MT-RNR1 | chrM:640-1601 |
| MT-TL1 | chrM:3230-3304 |
| MT-CO1 | chrM::5904-7445 |
| MT-TS1 | chrM:7446-7514 |
| MT-TK | chrM:8295-8364 |
| MT-TE | chrM:14674-14742 |

Table S2. **List of deafness-associated mitochondrial DNA regions**

Table S3. **List of deafness-associated microRNAs**

|  | |
| --- | --- |
| miR-96 | chr7:129414532-129414609 |
| miR-182 | chr7:129410223-129410332 |
| miR-183 | chr7:129414745-129414854 |
